# Surveillance of Mosquitoes (Diptera, Culicidae) in Kyiv, Ukraine Between 2013 and 2017

**DOI:** 10.1089/vbz.2020.2666

**Published:** 2021-02-24

**Authors:** Tata Romanenko, Natalyia Hunchenko, Tetiana Kharkhun, Lyudmila Kardupel, Larysa Honcharenko, Stephen Higgs

**Affiliations:** ^1^ State Institution Kyiv City Laboratory Center of the Ministry of Health of Ukraine, Kyiv, Ukraine.; ^2^ Biosecurity Research Institute, Kansas State University, Manhattan, Kansas, USA.

**Keywords:** surveillance, mosquitoes, Ukraine

## Abstract

For effective control of vector-borne diseases and control of nuisance-biting insects, it is important to know which species are present and their relative abundance. In this study, we report data from a State-supported mosquito surveillance program in Kyiv, Ukraine's capital city. The surveillance identified 29 different species: 24 Culicines and 5 Anopheline species. Culicine mosquitoes included 17 in the genus *Aedes*, 3 *Culex*, 3 *Culiseta*, and 1 *Mansonia* species. The relative abundance of each genera was consistent in years 2014, 2015, and 2016; namely *Aedes*>*Culex*>*Anopheles*. In 2017, *Aedes* and *Culex* mosquitoes were approximately the same, predominating over *Anopheles*. A declining trend in the numbers of mosquitoes collected from 2013 to 2017 has not only several potential explanations, including increased urbanization and more effective control, but also may reflect changes in surveillance efforts.

## Introduction

The recent emergence of several mosquito-borne viruses, including chikungunya and Zika viruses in the Northern hemisphere, has largely been attributed to international travel of infected people from endemic regions with ongoing transmission cycles and exacerbated by the spread of invasive mosquito species.

In Europe, a chikungunya outbreak was first reported in Italy (Rezza et al. 2007) and subsequently local transmission occurred in France (Grandadam et al. 2011). Human Zika cases have been reported in many European countries (Zanolli et al. 2018).

In Ukraine, several mosquito-borne viruses have been detected, including, Batai, Bunyamwera, Olyka, California encephalitis, Tahyna, and West Nile (Hubálek 2000, 2008, Napp et al. 2018). West Nile virus (WNV) emerged in Belarus and Ukraine during the 1970s and 1980s (Buletsa et al. 1989, Hubálek and Halouzka 1999, Gratz 2004) with more frequent detections since 2011 (Ziegler et al. 2013, Napp et al. 2018).

California encephalitis virus (CEV) was first detected in Ukraine in 1980s and 1990s (Lozyns'kyĭ and Vynohrad 1998). Batai virus and the Olyka strain have been isolated from mosquitoes in Ukraine, specifically from *Anopheles maculipennis* s.l. (Vinograd et al. 1973, Terekhin et al. 2010). Lozyns'kyĭ and Vynohrad (1998) reported serological evidence suggesting the presence of Inko and snowshoe hare virus. According to Hubálek (2008), antibodies to Sindbis virus have been reported from Ukraine, but no virus isolations have been made.

Nonarbovirus pathogens transmitted by mosquitoes in Ukraine include the filarial nematode worms *Dirofilaria immitis* and *Dirofilaria repens* (Kartashev et al. 2014). As experienced in many countries, because of a lack of approved vaccines, control of these vector-borne pathogens is primarily dependent on reducing vector populations and educating people about methods to minimize risk of exposure.

For effective control of mosquito-borne diseases, it is important to know which species of mosquitoes are present, their seasonal abundance, biology, and competence to transmit specific pathogens. This report describes a longitudinal surveillance study conducted by the Ministry of Health (MOH) personnel to identify species of mosquitoes in Kyiv, an urban environment that is a destination for many international travelers to Ukraine. In Ukraine, autochthonous cases of malaria were eliminated in 1956, however, imported cases are occasionally reported, and surveillance for potential vectors, as reported here, continues as a part of the program to evaluate risk of local transmission.

## Methods

**FIG. 1.**
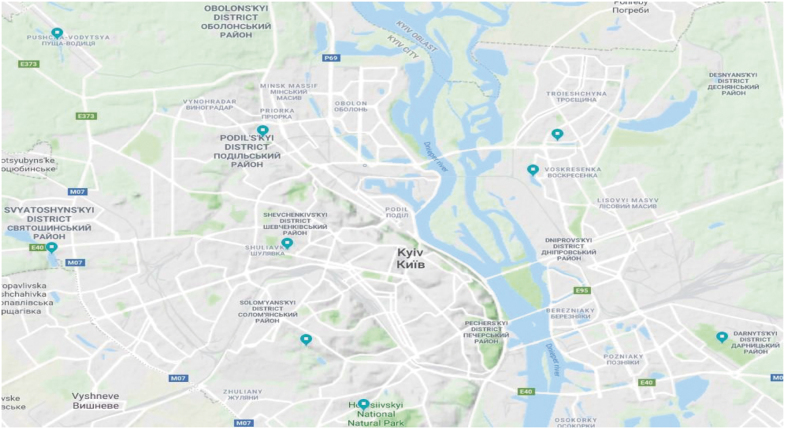

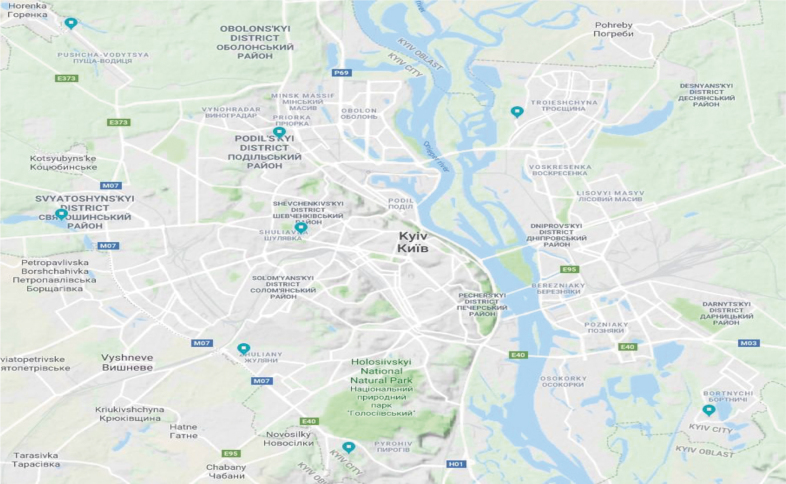
**(a)** Collection sites from which larvae were obtained. **(b)** Collection sites from which adult mosquitoes were obtained.

Mosquito larvae and adults were collected using well-established methods, including the use of nets and dippers (Service 1993). Collections were conducted between March and October of each year. Mosquito larvae were collected once per week with nets/dippers, from three reservoirs on the West bank of the Dnipro River and six reservoirs on the East bank (Fig. 1a, Table 1), all within the Kyiv city limits. Further details of the collection sites, including coordinates, are provided in Table 1. Larvae were classified to species based on morphological characteristic at the L4 stage using keys that have been written specifically for use in Ukraine (Sheremet 1998, Kilochytska 2008, Prudkina 2011). Population densities were approximated based on square meter of the water surface that was sampled.

**Table 1.** Collection Sites from Which Larvae Were Obtained

Districts of Kyiv/Paйони м.Києвa	Name/Haзвa	Address/Aдpeсa	Coordinates/Кооpдинaти
Holosiivskyi/Γолосіївський	The reservoir №3/Bодоймищe №3	Orikhuvat reservoirs in the Holosiivsky park named by Rylsky/Оpіхувaтські водойми в Γолосіївському пapку ім.Pильського	Bысотa: 151 meters N 50°23′11.247″ E 30°30′00.239″ N 50°23.187′ E 30°30.003′
Darnytskyi/Дapницький	Reservoirs №№1, 2, 3/Bодойми №№1,2,3	Park Partyzanskoi Slavy/ Пapк Пapтизaнської Cлaви	Bысотa: 106 meters N 50°24′49.960″ E 30°40′28.098″ N 50°24.832′ E 30°40.468′
Desniansky/Дeснянський	Water reservoirs on the Zakrevsky street/Bодойми кaнaлу по вул.Зaкpeвського	Zakrevsky street/ вул.Зaкpeвського	Bысотa: 94 meters N 50°29′48.003″ E 30°35′54.603″ N 50°29.800′ E 30°35.910′
Dniprovsky/Дніпpовський	The lake Radunka (Raiduzhne)/Озepо Paдункa (Paйдужнe)	Cheremshiny Street/вул.Чepeмшини	Bысотa: 94 meters N 50°28′53.130″ E 30°35′02.866″ N 50°28.885′ E 30°35.047′
Obolonskiy/Оболонський	The pond/Cтaвок	Pushcha-Vodytsia, 7 line/Пущa Bодиця, 7 лінія	Bысотa: 146 meters N 50°33′00.102″ E 30°21′54.863″ N 50°33.001′ E 30°21.914′
Podilsky/Подільський	Lake of the National Ecological and Naturalistic Center for Student Youth/Озepо Haціонaльного eколого-нaтуpaлістичного цeнтpу молоді	National Ecological and Naturalistic Center for Student Youth Vyshgorodskaya Street, 19/Haціонaльний eколого-нaтуpaлістичний цeнтp молоді вул.Bишгоpодськa, 19	Bысотa: 127 meters N 50°29′52.290″ E 30°27′11.646″ N 50°29.871′ E 30°27.194′
Svyatoshinsky/Cвятошинський	The reservoir №14/Bодоймищe №14	Verkhovyna Street/Bул.Bepховиннa	Bысотa: 134 meters N 50°27′00.947″ E 30°20′46.905″ N 50°27.015′ E 30°20.781′
Solomensky/Cолом’янський	The reservoir №3/Bодоймищe №3	Sovska Balka/Cовськa Бaлкa	Bысотa: 138 meters N 50°24′43.019″ E 30°28′28.961″ N 50°24.716′ E 30°28.482′
Shevchenkivsky Шeвчeнківський	The reservoir №7/Bодоймищe №7	Peremohy prosp.32/пp.Пepeмоги, 32	Bысотa: 150 meters N 50°27′08.226″ E 30°27′.248″ N 50°27.137′ E 30°27.787′

To collect adult mosquitoes, two methods were used. Once per month, three entomologists employed by the State Institution (SI), Kyiv City Laboratory Center of the MOH of Ukraine, collected adult mosquitoes from eight privately owned livestock-holding areas (two on the West bank and six on the East bank) (Fig. 1b, Table 2). Mosquitoes were collected from randomly selected 1 m^2^ quadrats on the walls and ceiling of the barn and placed into tubes for transfer to the laboratory to be speciated. The second collection technique used was the human landing method. The three entomologists collected mosquitoes that landed on them over a 20-min period. This method is well-established and frequently used for adult mosquito collection (Achee et al. 2015) and was reviewed and approved by a MOH Biosafety Committee. After transfer to the laboratory, mosquitoes were identified to species by entomologists using keys (Sheremet 1998, Kilochytska 2008, Prudkina 2011).

**Table 2.** Collection Sites from Which Adult Mosquitoes Were Obtained

Districts of Kyiv/Paйони м.Києвa	Address/Aдpeсa	Coordinates/Кооpдинaти
Holosiivskyi/Γолосіївський	Academica Lebedeva Street, 19/вул.Aкaдeмікa Лeбeдєвa,19	Bысотa: 178 meters N 50°20′47.453″ E 30°29′20.466″ N 50°20.790′ E 30°29.341′
Darnytskyi/Дapницький	Village Bortnichi, Mostova street, 40/сeло Боpтничі, вул. Mостовa, 40	Bысотa: 96 meters N 50°21′48.937″ E 30°41′05.814″ N 50°21.815′ E 30°41.096′
Desniansky/Дeснянський	Village Troyeshchyna, str. Karl Marx, 44/сeло Tpоєщинa, вул. Кapлa Mapксa, 44	Bысотa: 100 meters N 50°30′29.447″ E 30°34′48.151″ N 50°30.490′ E 30°34.802′
Obolonskiy/Оболонський	Pushcha-Vodytsia, 14 line/Пущa-Bодиця, 14 лінія	Bысотa: 134 meters N 50°33′03.022″ E 30°20′13.553″ N 50°33.050′
Svyatoshinsky/Cвятошинський	Brest-Lytovske highw. 17 Fisheries Nyvka/Бpeст-Литовськe шосe, 17, pибнe господapство “Hивкa”	Bысотa: 140 meters N 50°26′32.071″ E 30°20′38.659″ N 50°26.534′ E 30°20.644′
Solomensky/Cолом’янський	Shevchenko prov./пpов.Шeвчeнкa,18	Bысотa: 179 meters N 50°21′24.691″ E 30°4′13.291″ N 50°1.411′ E 30°4.221′
Podilsky/Подільський	National Ecological and Naturalistic Center for Student Youth Vyshgorodskaya Street, 19/Haціонaльний eколого-нaтуpaлістичний цeнтp молоді, вул.Bишгоpодськa, 19	Bысотa: 127 meters N 50°29′52.290″ E 30°27′11.646″ N 50°29.871′ E 30°27.194′
Shevchenkivsky Шeвчeнківський	Peremohy prosp.32/пp.Пepeмоги, 32	Bысотa: 150 meters N 50°27′08.226″ E 30°27′47.248″ N 50°27.137′ E 30°27.787′

## Results

Between 2013 and 2017, 11,741 adult mosquitoes were identified by entomologists of the SI, Kyiv City Laboratory Center of the MOH of Ukraine. The collection included 5272 potential Anopheline malaria vectors and 6460 Culicine mosquitoes. Culicine mosquitoes predominated the collections and constituted 55.1% of total number collected. The mosquitoes identified belonged to *Aedes*, *Culex*, *Culiseta*, and *Mansonia* genera. More species were collected on the Dnipro River East bank than on the West bank—24 species, and 13 species, respectively. A total of 24 different species of Culicinae mosquitoes were identified: *Aedes cantans, Aedes caspius caspius, Aedes caspius dorsalis, Aedes cataphylla, Aedes cinereus, Aedes communis, Aedes punctor, Aedes cyprius, Aedes behningi, Aedes detritus, Aedes diantaeus, Aedes excrucians, Aedes geniculatus, Aedes sticticus, Aedes vexans, Aedes pullatus, Aedes riparius, Culex pipiens pipiens, Culex pipiens molestus, Culex pipiens modestus, Culiseta annulata, Culiseta glaphyroptera, Culiseta alaskaensis,* and *Mansonia richiardii.*

In addition, five Anophelines were collected; *Anopheles atroparvus, Anopheles claviger, An. maculipennis maculipennis, Anopheles maculipennis messeae,* and *Anopheles plumbeus*.

Of these species, *Cx. pipiens*, *Ae. cantans*, *Aedes cataphyla*, *Ae. riparius*, and *Ae. sticticus* predominated in collections from the East bank; *Ae. pullatus*, *Ae. vexans*, *Ae. cinereus*, and *Ae. sticticus* primarily exist on the West bank. Of the Anophelines, only three were present on both banks of the river; *An. claviger*, *An. maculipennis maculipennis*, and *An. maculipennis messeae*.

**FIG. 2.**
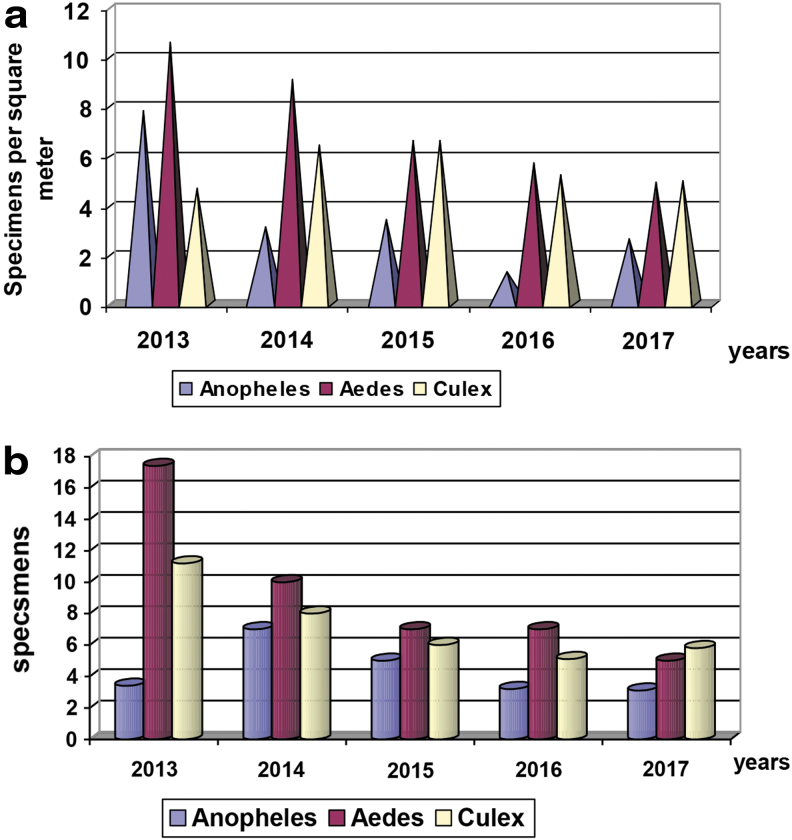
**(a)** The ASNI of mosquito larvae in Kyiv from 2013 to 2017 (specimens per square meter). **(b)** The ASNI of mosquito adults collected by various methods in Kyiv from 2013 to 2017. ASNI, average seasonal number index.

**FIG. 3.**
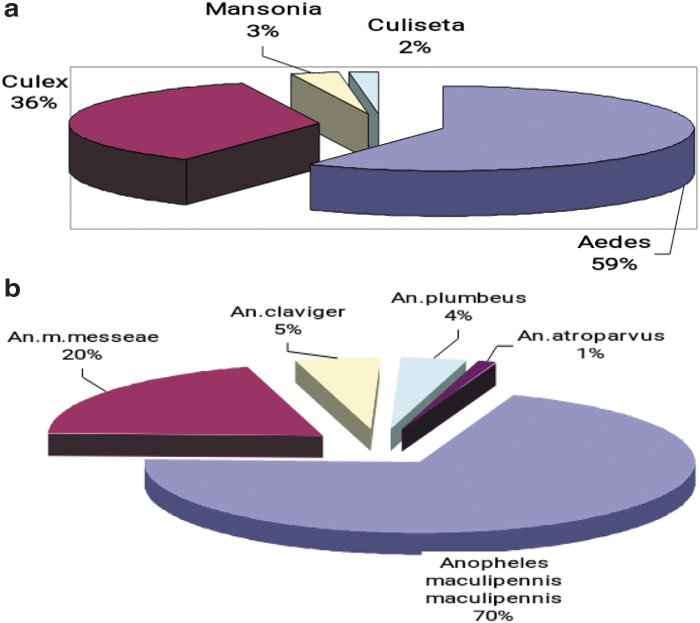
**(a)** The species composition of Culicine mosquitoes in Kyiv from 2013 to 2017. **(b)** The species composition of Anopheline mosquitoes in Kyiv from 2013 to 2017.

The average seasonal number index (ASNI) of *Aedes* mosquito larvae based on dipper collection, decreased by 2.2-fold in 2017 compared with 2013 (Fig. 2a). The combined ASNI number index of *Culex* mosquito larvae (collected with dippers and standard nets) increased by 6.4% in 2017 compared to 2013, but decreased by 3.8% compared to 2016. The ASNI of adult *Aedes* mosquitoes, based on human landing collection, showed a 3.5-fold decrease during the last 5 years: in 2013 ASNI was 17.4, and in 2017 was 5.0 (Fig. 2b). As shown in these figures, the relative abundance of each genera for both larvae and adults were consistent in years 2014, 2015, and 2016; namely *Aedes*>*Culex*>*Anopheles*. Although in 2013, larval *Anopheles* predominated over *Culex*, for adults, *Culex* predominated over *Anopheles*. In 2017, *Aedes* and *Culex* mosquitoes were approximately the same, predominating over *Anopheles*. If all data from 2013 to 2017 are combined, species of mosquitoes in the genus *Aedes,* represented 59% of Culicine mosquitoes whilst *Culex* species represented 36% (Fig. 3a).

Anopheline mosquitoes constituted 44.9% of the total. The predominant species that existed in the surveillance areas are *An. maculipennis maculipennis* and *An. maculipennis messeae* (Fig. 3b). On the East bank, five of seven known *Anopheles* species in Ukraine were identified, namely *An. maculipennis maculipennis*, *An. maculipennis messeae*, *An. claviger*, *An. plumbeus*, and *An. atroparvus*. Some species were only identified on the West bank: *An. maculipennis*, *An. maculipennis messeae*, and *An. claviger*. The ASNI of Anopheline mosquito adults (per square meter in stock building) in Kyiv decreased 2.3-fold: from 7.0 in 2014 to 3.1 in 2017. The ASNI of *Anopheles* larvae (registering with dippers or standard nets) increased twofold in 2017 compared to 2016, but decreased threefold in 2017 compared with 2013.

## Discussion

Our surveillance identified 29 different mosquito species. Data from all collection methods used showed a declining trend of mosquito population abundance from 2013 to 2016. A previous study (Kilochytska 2012) discussed 35 species of anthropophilic mosquito and environmental conditions in Kyiv.

The relative abundance of 10 species collected between 1992 and 2008 was evaluated, although their potential significance with respect to the transmission of pathogens was not discussed. There are several potential explanations for the observed changes in abundance over time and the distribution of different species reported in the current study. It is possible that increased urbanization, the seasonal (*i.e.*, April to October) mosquito control efforts that include insecticide spraying of buildings and efforts to clear vegetation from standing water, drainage, and cleanup activities at aquatic breeding sites may have resulted in a population decline.

Although urbanization may have a negative impact on the abundance and distribution of some mosquito species, as described by Kilochytska (2013), the availability of domestic premises may enable some species to increase.

With regard to our findings, reduced funding to support surveillance efforts and reorganization of State sanitation programs may have resulted in the perception of population reduction simply because less collecting effort results in fewer numbers of mosquito captures to report. In recent years, public awareness of mosquito-borne viruses has likely increased, and residents of Kyiv can submit information on, for example, nuisance biting by mosquitoes to a State website that can be used to enhance MOH surveillance and control efforts.

Different government entities exchange information to direct mosquito control operations and specialists at the SI Kyiv City Laboratory Center of the MOH of Ukraine annually develop comprehensive task plans for antimalarial and antiepidemic work. Despite the fact that the underlying cause of the reduced abundance from 2013 to 2016 cannot be conclusively determined, the surveillance data provide an understanding of mosquitoes in Kyiv, which are potential vectors of pathogens that can infect humans and other animals.

Similarly, the observed differences in species distribution at different collection sites cannot be fully explained since the exact conditions that promote one species, for example *Ae. vexans*, primarily on the West bank, and *An. atroparvus*, only on the East bank, are not known.

It cannot be assumed that the river itself is a physical barrier that controls mosquito distribution. Nonetheless, the knowledge of species distribution can be important to target and optimize control efforts to control those species, for example, *Cx. pipiens. Cx. pipiens molestus, Cx. pipiens modestus*, and *Ae. vexans* that are known to be efficient vectors of important arboviruses such as West Nile virus that, as described above, is present in Ukraine (Hubálek and Halouzka 1999, Napp et al. 2018).

Of the other species that were identified in this survey, *Ae. caspius* is a potential vector of West Nile virus and of California encephalitis virus, and *Cx. pipiens modestus* is also a potential vector of California encephalitis virus. For Batai virus, a potential vector is *Ae. communis*. This is also a vector of Inkoo virus. The aggressive human-biting *Ae. vexans* is regarded as a vector for Tahyna, as are *Ae. caspius*, *Ae. cantans*, *Ae. cinereus*, *Ae. sticticus*, and *Cx. pipiens modestus* (Medlock et al. 2007).

With respect to *Anopheles* spp., locally transmitted cases of malaria has not occurred in Ukraine since 1956. Imported cases of malaria are, however, reported every year. In 2017, 45 cases of malaria were imported to Ukraine: 80% attributed to *Plasmodium falciparum* (Andreychin et al. 2019). In Ukraine, *An. maculipennis* has been implicated as a potential vector for Batai virus and WNV (Gratz 2004).

An important finding from the survey was that, despite being a major international transport and trade hub in Ukraine, we did not find any evidence to indicate the presence of the highly invasive species *Aedes aegypti* and *Aedes albopictus*. These invasive species are potential vectors of several important viruses that infect humans, including chikungunya, dengue, and Zika (Medlock et al. 2012).

In conclusion, in view of human migration and tourism, continuing urban development, and international trade, we recommend continued entomological monitoring for mosquitoes in Kyiv to determinate the species composition in the city. Such surveillance is critical to prepare for introductions of nonnative species and possible transmission of travel associated vector-borne pathogens.

## References

[B1] Achee NL, Youngblood L, Bangs MJ, Lavery JV, et al. Considerations for the use of human participants in vector biology research: A tool for investigators and regulators. Vector Borne Zoonotic Dis 2015; 15:89–102 25700039 10.1089/vbz.2014.1628 PMC4340630

[B2] Andreychyn M, Kopcha V, Ishchuk I, Iosyk I. Imported tropical malaria (case report). Georgian Med News 2019; 294:109–113 31687960

[B3] Buletsa BA, Turak JA, Korol MJ, Ignatovich II, et al. Neurological manifestations of West Nile fever in the Transcarpathian region, Ukrainian SSR [in Russian]. Zh Nevrol Psikhiatr 1989; 89:29–30 2543166

[B4] Grandadam M, Caro V, Plumet S, Thiberge JM, et al. Chikungunya virus, southeastern France. Emerg Infect Dis 2011; 17:910–913 21529410 10.3201/eid1705.101873 PMC3321794

[B5] Gratz NG, World Health Organization (WHO). The vector-borne human infections of Europe: Their distribution and burden on public health. WHO Regional Office for Europe 2011. 2004. Available at https://apps.who.int/iris/handle/10665/107548

[B6] Hubálek Z. European experience with the West Nile virus ecology and epidemiology: Could it be relevant for the New World? Viral Immunol 2000; 13:415–426 11192288 10.1089/vim.2000.13.415

[B7] Hubálek Z. Mosquito-borne viruses in Europe. Parasitol Res 2008; 103(Suppl 1):S29–S43 19030884 10.1007/s00436-008-1064-7

[B8] Hubálek Z, Halouzka J. West Nile fever—A reemerging mosquito-borne viral disease in Europe. Emerg Infect Dis 1999; 5:643–650 10511520 10.3201/eid0505.990505 PMC2627720

[B9] Kartashev V, Afonin A, González-Miguel J, Sepúlveda R, et al. Regional warming and emerging vector-borne zoonotic dirofilariosis in the Russian Federation, Ukraine, and other post-Soviet states from 1981 to 2011 and projection by 2030. Biomed Res Int 2014; 2014:858936 25045709 10.1155/2014/858936 PMC4090463

[B10] Kilochytska N. A brief determinant of the bloodsucking mosquitos of the fauna of Ukraine, Kyiv. 2008:10

[B11] Kilochytska NA. Extension of habitat of female blood-sucking mosquitoes in Solomenskiy district, Kiev [In Ukrainian with English summary]. Visnyk Dnipropetrosk Univ Biol Med 2013; 4:71–75

[B12] Kilochytska NP. Synanthropy of bloodsucking mosquitoes (Diptera, Culicidae) under conditions of Kyiv. Vestnik Zoologii 2012; 46:e15–e20

[B13] Lozyns'kyĭ IM, Vynohrad IA. Arbovirusy ta arbovirusni infektsiï u lisostepoviĭ zoni Ukraïny [Arboviruses and arbovirus infections in the forest steppe zone of Ukraine]. Mikrobiol Z 1998; 60:49–60 9670754

[B14] Medlock JM, Hansford KM, Schaffner F, Versteirt V, et al. A review of the invasive mosquitoes in Europe: Ecology, public health risks, and control options. Vector Borne Zoonotic Dis 2012; 12:435–447 22448724 10.1089/vbz.2011.0814 PMC3366101

[B15] Medlock JM, Snow KR, Leach S. Possible ecology and epidemiology of medically important mosquito-borne arboviruses in Great Britain. Epidemiol Infect 2007; 135:466–482 16893487 10.1017/S0950268806007047 PMC2870593

[B16] Napp S, Petrić D, Busquets N. West Nile virus and other mosquito-borne viruses present in Eastern Europe. Pathog Glob Health 2018; 112:233–248 29979950 10.1080/20477724.2018.1483567 PMC6225508

[B17] Prudkina N. The bloodsucking Diptera insects. Kharkov 2011; 20–21

[B18] Rezza G, Nicoletti L, Angelini R, Romi R, et al. “Infection with chikungunya virus in Italy: An outbreak in a temperate region.” Lancet 2007; 370:1840–1846 18061059 10.1016/S0140-6736(07)61779-6

[B19] Service MW. Sampling the larval population. In: Mosquito Ecology: Field Sampling Methods, 2nd ed. Chapman & Hall, UK, Elsevier Science Publishers Ltd., 1993: 75–209

[B20] Sheremet V. *The Bloodsucking Mosquitos of Ukraine*. Tutorial. EPC National University of Kyiv 1998:12

[B21] Terekhin SA, Grebennikova TV, Khutoretskaia NV, Butenko AM. Molecular-genetic analysis of the Batai virus strains isolated from mosquitoes in Volgograd region of the Russian Federation, West Ukraine, and Czech Republic [in Russian]. Mol Gen Mikrobiol Virusol 2010; 27–29 20364478

[B22] Vinograd LA, Gaĭdamovich SIa, Obukhova VR, Vigovskiĭ AI, et al. Izuchenie biologicheskikh svoĭstv virusa Olyka, vydelennogo ot komarov (Culicidae) na Zapade Ukrainy [Study of the biological properties of the Olyka virus isolated from mosquitoes (Culicidae) in the western Ukraine]. Vopr Virusol 1973; 18:714–719 4151318

[B23] Zanolli S, Morotti M, Denicolo A, Tassinari M, et al. Chikungunya virus and Zika virus in Europe. In: Higgs S, Vanlandingham DL, Powers AM eds. *N Chikungunya and Zika Viruses: Global Emerging Health Threats.* 1st ed. Elsevier Academic Press, 2018:193–214

[B24] Ziegler U, Skrypnyk A, Keller M, Staubach C, et al. West Nile virus antibody prevalence in horses of Ukraine. Viruses 2013; 5:2469–2482 24100889 10.3390/v5102469 PMC3814598

